# Psychosocial Interventions for the Treatment of Cancer-Related Fatigue: An Umbrella Review

**DOI:** 10.3390/curroncol30030226

**Published:** 2023-03-01

**Authors:** Nieves Cedenilla Ramón, Jose Ignacio Calvo Arenillas, Sandra Aranda Valero, Alba Sánchez Guzmán, Pedro Moruno Miralles

**Affiliations:** 1 Department of Psychology, University of Castilla la Mancha, 45600 Talavera de la Reina, Spain; 2 Doctoral School “Studii Salamantini”, University of Salamanca, 37008 Salamanca, Spain; 3 Department of Physics, Engineering and Medical Radiology, University of Salamanca, 37008 Salamanca, Spain; 4 Department of Occupational Therapy, Association of Families of People with Intellectual and Developmental Disabilities of Toledo (APANAS), 45003 Toledo, Spain; 5 Occupational Therapy Department, Virgen de Fuentes Claras Residence Valverde de la Vera, 10490 Cáceres, Spain; 6 Department of Nursing, Physiotherapy and Occupational Therapy, University of Castilla la Mancha, 45600 Talavera de la Reina, Spain

**Keywords:** cancer, oncology, fatigue, treatment, “psychosocial interventions” and reviews

## Abstract

Cancer-related fatigue is one of the most common symptoms of cancer and one of those referred by patients as the most disabling. However, we still do not have enough evidence to allow us to recommend effective and personalized approaches. Goal: To provide evidence on the efficacy of ASCO-recommended psychosocial interventions for reducing cancer-related fatigue. Methodology: A general quantitative systematic review for nonprimary clinical interventions that allows the collection, synthesis and analysis of already published reviews. Systematic reviews of RTCs were selected as these make up the body of knowledge that provides the most evidence in an umbrella format. The results do not provide clear or comparable evidence regarding the different interventions, with moderate evidence standing out for cognitive interventions and mindfulness. Conclusions: Research gaps, study biases and the need for further research to ask more precise questions and to make reliable recommendations to mitigate the impact of cancer-related fatigue are evident.

## 1. Introduction

Cancer is a set of pathologies whose high incidence has remained stable in recent years in most of the Western world. This fact promotes ongoing research related to medical intervention protocols, which has favoured higher survival rates, approximately 52.5% worldwide, according to data from the International Agency for Research on Cancer in 2018, and approximately 61.7% in women and 55.3% in men in countries with a high life expectancy, such as Spain, according to data from the Spanish Society of Medical Oncology (hereafter SEOM) [1]. However, the increase in survival also results in individuals with a significant deterioration in health-related quality of life (HRQL from now on), due to numerous side effects in the short, medium and long term, both physical and psychosocial, in affected individuals [2,3,4,5,6,7,8]. Maintaining quality of life is an important goal of cancer treatment [9,10,11,12,13].

Among the conditions associated with cancer that have the greatest impact on the HRQoL of patients, we find fatigue [14,15,16,17,18,19,20]. According to the literature, we can affirm that cancer-related fatigue (hereafter CRF) is one of the most common symptoms and also one of those referred by patients as the most disabling [21,22,23,24,25]. Fatigue is not only a prevalent symptom but also a factor that affects patients’ quality of life.

Based on this evidence, already in 2014, the American Society of Clinical Oncology (ASCO, hereinafter), proposed a set of recommendations for the detection, evaluation and management of fatigue in survivors of cancer [26]. Among their recommendations is the development of interventions based on physical activity, psychosocial interventions, education and counselling and mind–body interventions. Recommendations were reviewed and validated two years later [27].

These recommendations confirm a known reality, although not always considered; the implications, both in the short and long term, of primary cancer treatments on the HRQoL of survivors are often not evaluated. Consequently, they are not always treated, as is the case with CRF. The absence of intervention on these aspects means that cancer, in addition to being a serious health problem, has an important social, economic and human impact in the short, medium and even long term [28,29,30,31,32,33,34].

On the other hand, the pathogenesis of CRF is not well identified and may be related to multiple and diverse factors that may contribute to its development and maintenance [35,36]. Among the possible factors are medical protocols. Biological and hormonal therapies have been especially well documented, which are associated with more severe and persistent fatigue experiences of a penetrating and cumulative nature [35,36,37,38]. However, these studies do not satisfactorily explain the persistent fatigue so commonly documented in survivors [39], since this is related to traits and characteristics of all patients. In summary, the importance of developing effective personalized approaches to reduce CRF is unquestionable.

Psychosocial interventions seem promising for the management of fatigue among survivors. For this review, psychosocial interventions are defined as various kinds of interventions provided to influence or change cognitions, emotions, behaviours, social inter-actions or a combination of these in order to achieve better mental health and/or fewer problems, including, among others, less fatigue [9,40].

However, the heterogeneity of disciplines and the types of interventions and outcome measures found in the literature on the efficacy of nonpharmacological treatments in reducing FRC does not allow us to conclusively identify which type of intervention is the most effective. Consequently, there is no certain way to select intervention recommendations consistently and based on the available scientific evidence. This diversity of research makes a detailed analysis of published studies necessary to identify the most effective treatments among all those found in the literature. Therefore, we believe that the development of this research is fully justified.

The objective of this review was to search, evaluate and synthesize the scientific evidence on psychosocial interventions to reduce CRF in cancer survivors. The research questions that guided the review were designed in such a way as to be flexible enough to identify any available and accessible intervention or treatment to reduce FRC in cancer survivors. The questions that this review aims to answer are as follows:

(a) What is the volume and characteristics of research on psychosocial treatments for the reduction of cancer-related fatigue?
(b) What psychosocial interventions or treatment modalities present evidence of efficacy for the reduction of cancer-related fatigue?

## 2. Materials and Methods

An umbrella review method proposed by Aromataris [41] was used to synthesize the evidence of clinical interventions in CRF. An umbrella review’s most characteristic feature is that this type of evidence synthesis only considers for inclusion the highest level of evidence, namely other systematic reviews and meta-analyses, not primary clinical studies. Therefore, an umbrella review allows the findings of reviews relevant to a review question to be compared. For these reasons, the broad picture obtained from the conduct of an umbrella review is ideal to highlight whether the evidence base around interventions in cancer-related fatigue is consistent or contradictory and to explore the reasons for the findings.

This type of methodology was selected as there are not enough studies with a comparable methodology to allow a meta-analysis to be carried out and in order to obtain more precise estimates of the overall effects derived from the individual studies included in the reviews [41,42]. In this way, it is possible to analyse a broad body of knowledge, both in terms of disciplines and research methods, to provide scientific evidence for decision makers in healthcare generally, and oncology specifically, to gain a clear understanding of the treatment of this symptom.

This umbrella review was preceded by an a priori, peer-reviewed protocol (NCR and PMM), not registered, with the following phases: (a) the identification and collection of reviews, (b) detailed inclusion criteria, (c) a formal process of data extraction, and (d) method for critical appraisal of the included reviews followed by the means to summarize and present the data [43].

### 2.1. Identification and Collection of Articles

First, a search strategy was created (11 December 2019) for Medline. The formal search string was (TITLE-ABS-KEY (cancer) OR TITLE-ABS-KEY (onco*) AND TITLE-ABS-KEY (fatigue) AND TITLE-ABS-KEY (treatment) OR TITLE-ABS-KEY (psychosocial interventions) AND TITLE-ABS-KEY (reviews). This was adapted to the rest of the databases consulted. In this way, it was established whether the terms contained in the title, abstract or keywords of the retrieved citations were associated with the planned search terms. Second, the formal literature search was performed in the following selected databases: Web of Science, Scopus, MEDLINE and SciELO. The search was carried out by title, abstract and keywords during the period from 2000 to 2021 for papers written in English and Spanish. A total of 2682 entries were identified; this was updated in February 2022.

With this search strategy, we attempted to collect information as completely as possible, incorporating a selection of databases that had not been used in previous reviews. Our objective was to avoid any bias that could diminish the quality of the information obtained.

After the process of identification and the screening of articles was repeated in the different sources, the titles and abstracts were read independently by two reviewers (N.C.R. and A.S.G.). To determine the eligibility of the articles, an “Eligibility checklist” was used, prepared by the research team. Four of the articles were not located.

Once a consensus between the reviewers was reached, articles were obtained to be reviewed in full. After reading the full texts, articles were eliminated because they did not meet the eligibility criteria. Finally, the entire article selection process was supervised independently by a third reviewer (P.M.M.).

In this phase, the researchers reviewed the full-text versions of the articles, and these were retained if they met the criteria. Agreement on inclusion and exclusion allocation was unanimous.

### 2.2. Detailed Inclusion Criteria

The following eligibility criteria were established according to the research question (PICO questions; P: cancer survivors; I: nonpharmacological interventions for the reduction of FRC; C: effectiveness of cognitive and behavioural psychosocial interventions, mindfulness, emotional undoing, etc.; O: reduction of cancer-related fatigue):

(a) Inclusion criteria: Reviews of the literature published in indexed journals, whose main objectives are to evaluate the efficacy of nonpharmacological psychosocial interventions for the reduction of FRC in cancer survivors. The interventions being compared are interventions based on cognitive models; emotional expression interventions, including art therapy; interventions based on psychoeducational strategies; interventions based on behavioural strategies; and mindfulness. We considered any measurement of outcome or effectiveness in reducing CRF. We included reviews with studies of Level 1b (well-designed individual randomized controlled trials—not a pilot or feasibility study with a small sample size) or Level 2b (individual prospective cohort studies, low-quality randomized controlled trials, e.g., <80% follow-up or low number of participants, pilot or feasibility studies; ecological studies; two-group, non-randomized studies).
(b) Exclusion criteria: Studies on the efficacy of drugs or nutritional and related supplements. Studies on the efficacy of exercise or variants of physical activity. Studies on the efficacy of minimally invasive or manipulative therapies. Studies that did not have the reduction of FRC as their main objective. Articles in which the keywords did not appear. A second publication of the same study.

### 2.3. Identification and Collection of Data

The 11 articles were registered and organized into a dynamic Excel^®^ 2016 worksheet designed by the research team, including the following items: publication data (title, authors, year, type of publication, journal/publisher, country, affiliation and language), intervention included in each review (objectives interventions, eligibility criteria, control group, intervention strategy, number of studies and types of studies), participant details (cancer type, age group, study sample and active treatment), study methodology (systematic reviews or meta-analysis), study design included in each review (RCTs, quasi-experimental, observational, cohort, case study, intervention projects or guidelines), study quality (PRISMA), intervention characteristics included in each review (type and subtype, duration, follow-up, sample and setting and context), quantitative and qualitative fatigue outcomes (instrument used to appraise the primary studies and method of synthesis/analysis used to synthesize the evidence), conclusions and other data of interest.

Subsequently, data were extracted from each study and included in the data extraction form. The data extraction process was carried out by researchers N.C.R. and S.A.V. independently. The extracted data were tabulated following an iterative process and using the categories designed for this study. Finally, the entire article registration process was supervised by a third reviewer (P.M.M.).

Next, a clustering process was carried out, exploring relationships in the data (differences and similarities) within and between studies to identify a common set of characteristics. This analysis considered the heterogeneity of the studies (especially study design, outcome measures of effectiveness, population and period of training), the risk of bias (any type of bias) and the internal validity of the studies. Our purpose was to analyse the characteristics of the studies to carry out a detailed critical review. The principal aim of an umbrella review is to provide a summary of existing research syntheses related to a given topic or question, not to resynthesize.

### 2.4. Methods for Critical Appraisal of the Included Reviews

Finally, an assessment of the methodological quality of the selected reviews was carried out. PRISMA [44] was used to assess the methodological quality of the reviews. The PRISMA application was carried out by two of the researchers in this study (N.C.R. and S.A.V.) independently. Subsequently, the evaluation process was reviewed by a third investigator (P.M.M.). Once the consensus of the reviewers was reached, these data were incorporated into the dynamic Excel^®^ table.

Additionally, the information provided by the authors of the reviews on the evaluation of the level of scientific evidence and the level of certainty is reported, as well as the risk of bias of the studies included in each review (see Appendix A).

In addition, we assessed clinical diversity by documenting participant characteristics represented in each study with a focus on factors, such as population, cancer type, survivors, study inclusion criteria and intervention control.

Furthermore, we documented heterogeneity in psychosocial interventions, such as temporality, specific fatigue, guidance, delivery mode and type of delivery and outcome measure. In addition, we assessed the diversity among ways of measuring fatigue and timing fatigue assessment (see Appendix B).

## 3. Results

**Figure 1:**
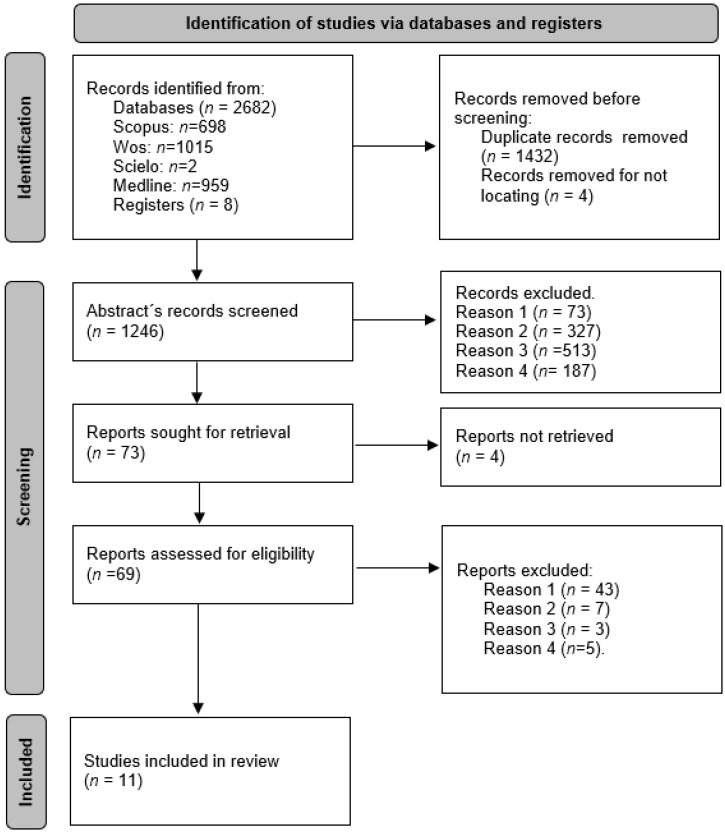
Flow diagram PRISMA 2020 (exclusion criteria: Reason 1—not collecting RTCs in the analysis. Reason 2—no CRF data in the analysis of results. Reason 3—noncancer-specific fatigue analysis. Reason 4—inclusion of treatments related to the administration of different substances or nutrients).

The search returned a total of 2682 results, of which 1432 were duplicates. After a first reading of the title and abstract, 73 articles were established as appropriate for retrieval. After applying the eligibility criteria, 69 articles were analysed in full. After reading the full texts, 58 articles were eliminated because they did not meet the eligibility criteria (see Figure 1). Finally, 11 reviews were included in the synthesis.

The main objective of this overview review was to collect and provide an abstract synthesizing the research evidence; therefore, the results are presented under three headings: Section 3.1) the volume and characteristics of the included reviews; Section 3.2) the methodological quality and critical analysis of the included reviews; and Section 3.3) the synthesis of research evidence.

### 3.1. Volume and Characteristics of Research

**Figure 2:**
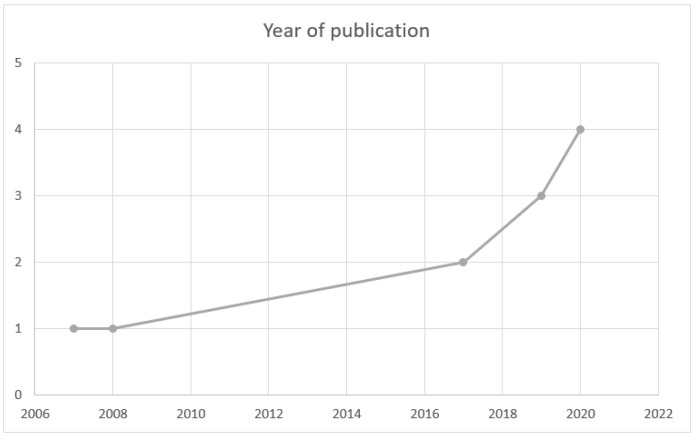
Year of publication.

Since 2006, a gradual and steady increase can be observed in the number of studies published. It should be noted that 81.8% of the reviews were published in the last five years, doubling the number of studies from 2017 to the present (Figure 2).

The countries of affiliation of the reviews analysed were China (45.5%), the USA (27.3%), the Netherlands (18.2%) and the United Kingdom (9.1%). It is worth noting that all the articles are affiliated with university institutions.

**Figure 3:**
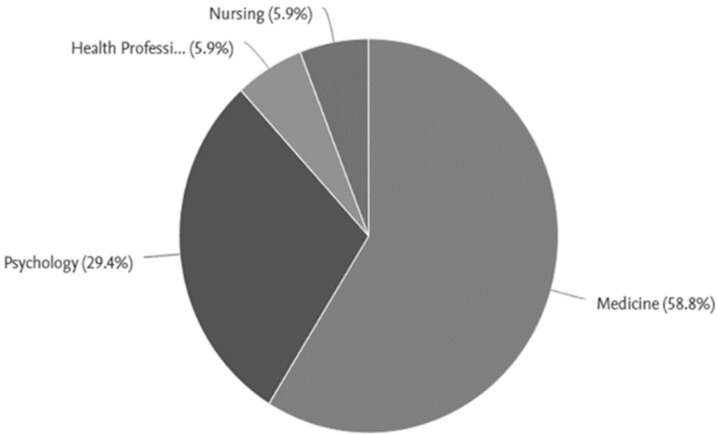
Knowledge areas.

With respect to the country of affiliation, the institutional status of the authors and co-authors was considered at the time of the publication of the article. All the articles were affiliated with university institutions. With regard to the area of knowledge, medicine (58.8%) and psychology (29.4%) were the most relevant, followed by nursing (5.9%) and other healthcare professions (5.9%) (Figure 3).

The total sample size of the RTCs included in the reviews ranged from 535 to 4525 (mean = 1922; median = 1603). The number of studies analysed in the reviews varied between 5 and 41 (mean = 16.9; median = 13). Regarding the populations studied, reviews of more than one type of cancer (81.8%) in adult cancer survivors, aged over 18 years, predominated, without considering the severity of the disease or the type of cancer (Table 1).

Six types of differentiated psychosocial interventions (psychosocial intervention is a generic way to refer to those whose objective is to influence or change cognitions, emotions, behaviours or a combination of these) have been identified: (a) Cognitive therapy (this category includes the following: psychological adjustment and support programs, problem solving therapy, cognitive behavioural therapy, social cognitive therapy, patient discussion, transtheoretical model, depression therapy, coping skills training and psychological support, motivational interviewing, imagery-based intervention, group relaxation and guided imagery and psychosocial support) (32.5%); (b) behavioural therapy (this category includes coping skills intervention; lifestyle, symptoms and health self-management; imagination-based behavioural intervention; brief behavioural-oriented interventions; and energy conservation and activity management intervention programs) (24%); (c) facilitating emotional expression therapies (this category includes programs of art therapy, expressive writing, movement therapy, dance, music therapy, emotion grooming and supportive expressive group therapy) (12%); (d) psychoeducational strategies (this category includes psychoeducational strategies for self-management without specifying facilitated techniques) (6.5%); (e) mindfulness (this category includes cognitive-based mindfulness techniques) (15.5%); and (f) others (this category includes miscellaneous programs, such as dyadic therapy, programs based on physical activity or rehabilitation exercises) (9.5%). The authors’ reports of the characteristics of primary sources are summarized in Appendix B.

It should be noted that only 18.5% of interventions are specifically designed to reduce FRC. A total of 47% are generic interventions with the potential influence to reduce FRC. Likewise, the duration of the interventions varied from less than one week to one year (mean = 9.6 weeks), with the number of sessions ranging from 1 to 52 sessions (mean of 9.4), mainly individually (46.4%) or as a group (37.3%), carried out by professionals in person (24.7%).

**Figure 4:**
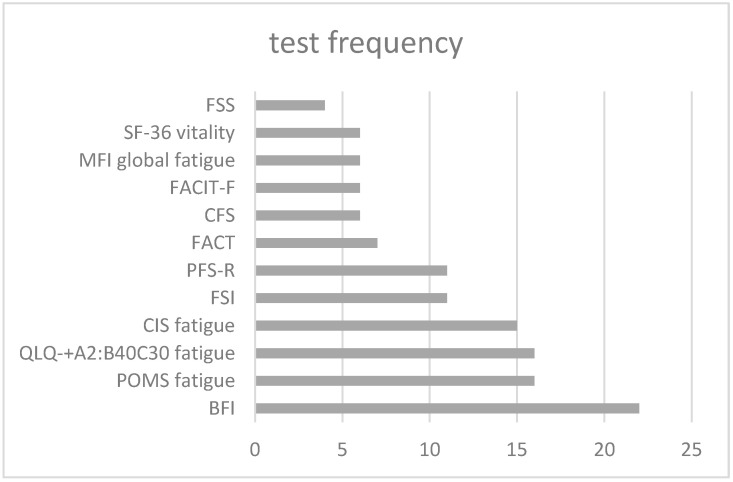
Test frequency.

Regarding the outcome measures, fatigue was evaluated in the eleven reviews (100%) as the main measure, although some of the reviews also presented other main and secondary results. The tools collected to assess fatigue were multiple. A total of 42 tools in the 173 trials were identified in the literature. Of note was the Brief Fatigue Inventory (BFI) used on 22 occasions (12.7%); QLQ-C30 fatigue and the Profile of Mood State Fatigue Subscale (POMS F), used in 16 studies (9.2%); CIS fatigue used in 15 studies (8.7%); and Fatigue Symptom Inventory (FSI) and Piper Fatigue Scale (PFS-R) used in 11 studies (6.4%). All other tools had less than 17% representation. A large group of tools (30.4%) were only used in a single study to complement other measures (see Figure 4).

### 3.2. Methodological Quality and Critical Analysis of Reviews

Table 2 provides summaries of the methodological quality of reviews according to PRISMA. The mean score was high (22.6), ranging between 19 and 26 points (see Table 2). 

The authors’ reports on the methodological quality of the primary sources are summarized in Appendix A. According to the levels of evidence reported, eight reviews were ranked as Level of Evidence 2B, due to low-quality RTCs [9,45,46,47,48,49,50,51]. Two of the reviews were ranked as Level 2B due to heterogeneous RTCs [52,53]. Finally, one of the reviews failed to report details of its evidence level [54]. The grade of recommendation is B.

Regarding the certainty levels of the evidence reported, four of the reviews (36.36%) refer to moderate levels of certainty regarding the estimation of the effect [46,47,50,51]. Four of the reviews (36.36%) refer to a low level of certainty; that is, the confidence level in the effect estimate is limited [45,48,49,53]. One of the reviews (9.09%) reports a high level of certainty; namely, there is much confidence that the true effect is close to the estimate [52]. One of the reviews (9.09%) reports very low quality of evidence [9].

In relation to the risk of bias for randomized trials included in each systematic review, the reported results indicate that performance biases, i.e., the blinding of participants and personnel and bias due to deviations from intended interventions, are the most significant biases, usually due to the impossibility of blinding the participants and the particularities of cancer patients (RoB 2) [55]. Next in importance are attrition biases and reporting biases, which do not allow the establishment of conclusive levels of results with some concerns. Within the primary studies of the reviews, sixty-six of the RTCs (12.34%) point out some concerns in relation to their risk of bias. Only one primary source (0.19%) reports a high score. Ten of the RTCs (1.87%) scored moderately. The rest of the primary sources (85.6%) are reported as low or without sufficient information.

### 3.3. Synthesis of Research Evidence

The direct and indirect effect sizes of the reviews were extracted [41], as well as the authors’ conclusions. Cognitive therapies [45,50,56] and, specifically, mindfulness [52,53] are those that report the best results in direct data in primary studies (Table 1). Better results are found in specific treatments in patients with previous fatigue [45,51].

Higher levels of evidence and strength of evidence indicate that cognitive behavioural therapy benefits female breast cancer patients with clinically relevant fatigue [45]. Likewise, coping skills training and psychological support strategies focused on perpetuating factors of postcancer fatigue [50,56,57,58] benefit adult survivors of cancer without specifying the type.

The MBSR intervention protocols studied in the reviews [50,52,53] present a methodological homogeneity that facilitates the evaluation of results and reasonable levels of quality. The three reviews indicate a significant effect on fatigue, even when they indicate a low or moderate level of certainty of the evidence due to the heterogeneity of other potentially confounding variables [50,53], such as the heterogeneity of outcome-measured tools or the different population profiles. The review with strong evidence is the one with the largest sample in a single protocol [52].

CRF-specific mindfulness in survivors older than 18 years introduced significantly greater improvements in fatigue interference than wait-list controls. The magnitude of the effect of MBSR on this and other fatigue outcomes, including fatigue severity, was significant at the end of the intervention and was maintained for at least 6 months [59]. These data are repeated in specific MBSR programs for MBSR(BC) cancer, and its symptoms [60] demonstrated greater improvement in fatigue. Most improvements in fatigue occurred during the training with little change occurring during the follow-up period.

In relation to the heterogeneity that allows the assessment of to what extent the results from different studies can be summarized in a single measure, the nine meta-analyses present scattered data. Eight of the meta-analyses use I^2^ with moderate mean heterogeneity, with values between 23% and 98% (median = 78.23; mean 69.18). One of the meta-analyses presents the test Q_w_ = 30.49 *p* < 0.05. The POLARIS project does not collect information on statistical heterogeneity, presenting data on possible moderating effects [61]. The two systematic reviews do not present heterogeneity data.

As highlighted by the authors, two (18.2%) of the reviews refer to significant evidence [49.52] without specifying the levels of heterogeneity of the primary studies. Five studies (45.5%) present effective results or some evidence [46,48,49,52,53] with heterogeneity data I^2^ = 82%; I^2^ = 87.46%; I^2^ > 50%; I^2^ = 93% and I^2^ = 93%. Two (18.2%) show limited support for intervention programs [51,54] with low-power heterogeneity statistics or no data. Two (18.2%) reports found no evidence of improvement in fatigue in the interventions analysed [9,45] with heterogeneity data at follow-up but not between interventions or without data.

## 4. Discussion

The growing population of cancer survivors justifies the urgent need to define effective interventions that optimize the functionality and quality of life of this population. Of the various types, interventions for the management of CRF-associated symptoms during cancer treatment and thereafter are some of the most important challenges facing oncology teams. This review provides a comprehensive assessment of the scientific evidence for the effectiveness of psychosocial interventions in reducing FRC in cancer patients.

First, the results of the primary studies reviewed indicate that cognitive behavioural techniques [45,50,51] and mindfulness [50,52,53,62] for CRF are the psychosocial interventions able to reduce CRF with greatest scientific evidence. This result is corroborated by international recommendations [27,35] as well as numerous primary studies. However, the strength of evidence for cognitive behavioural techniques for reducing FRC is moderate or low. Mindfulness protocols show strong evidence where there is methodological quality. They also point out that face-to-face guided interventions by trained therapists [48] are more effective than a limited number of self-directed, online sessions with non-specialized therapists.

Secondly, it should be noted that there is significant variability in the type of studies analysed. The characteristics of the studies included in the reviews selected in this research suggest that there is not enough consensus regarding the criteria to describe and classify the different aspects of psychosocial interventions [63] for CRF. This fact makes it difficult to compare the results of the different reviews and between their primary studies [43]. In different reviews, heterogeneity is observed in clinical, methodological and statistical data.

Many studies have evaluated the efficacy of interventions without considering the specific mechanisms of action of the different techniques or without these being specifically designed to control CRF. We identified a limited number of studies investigating interventions specifically aimed at addressing fatigue. It should also be noted that the efficacy of generic psychosocial techniques, such as relaxation or emotional relief, has been evaluated without focusing on the mechanisms of action or maintenance of the CRF.

In addition, the diversity of assessment tools and objective measures of CRF makes it difficult to indicate an unequivocal effect of psychosocial interventions in reducing CRF, as well as to combine individual results from each study to obtain a pooled estimate of effect without cause for confusion or ambiguity. Combinations of quantitative data and qualitative analysis, or the descriptive data narrative of the same, are commonly reported results.

We must also consider that the temporal extension, intensity and frequency of the programs evaluated may condition the interpretation of the results of the reviews analysed. The form of delivery seems to have a direct influence on the CRF reduction results. Current research evidence suggests that therapist-guided face-to-face programs were more effective than self-guided programs [61]. We believe self-guided interventions [47,48,49] or interventions with minimal contact with the professional [47,48] integrated with findings from individual face-to-face sessions [9,45,47,48,49,50,51,54] may call into question the results obtained in certain selected reviews.

In addition to this is the importance of the adherence to treatment and the long-term follow-up of the population with CRF [64]. None of the reviews analysed established follow-up periods greater than one year. In the included reviews, most trials integrated measures of the short- or medium-term (below 6 months) effectiveness of the different interventions on FRC, so the results of the actual effect on FRC reduction could be overestimated. CRF is documented as a chronic symptom and it would be advisable to include longer-term follow-up periods [12,65] because the long-term intervention could better demonstrate its real effect on fatigue management.

Third, in relation to the methodological quality of the reviews, the PRISMA scores show that the reviews were designed with moderate or high methodological quality. However, the inclusion of meta-analyses attempting to unify results may be questionable.

The objective of a systematic review and a meta-analysis is to be able to combine its results quantitatively in order to obtain evidence, and its success will depend on the presence and magnitude of heterogeneity [66]. If its value exceeds more than 50%, the recommendation is not to combine the individual results to obtain a summary measure, except to obtain an aggregate of biases from many different sources. In this case, a narrative synthesis would be more honest, as some authors indeed perform.

In the primary studies, they appear as high risk, especially regarding the impossibility of blinding and the distribution of the sample. Performance biases are due to deviations from intended interventions and the particularities of cancer patients. Attrition bias and reporting bias also do not allow the establishment of conclusive levels of results with some concerns [67,68].

On the other hand, a significant number of the control groups established in the various studies did not have any supervision regarding the amount of attention that each group received. In addition, the quality of the different trials included in the reviews was rated as low to moderate, largely due to limitations in study design. However, regarding the risk of bias between primary studies, this information is lacking or unclear in most reviews. It would be interesting to explore the unreported publication bias if it exists, considering the affiliation or editorial lines of the journals, as well as the countries of reference in the CRF study.

All the reviews used tools to assess the risk of bias and quality of evidence for their primary interventions. The most widely used tool was the Cochrane risk of bias (RoB) and GRADE profile. In general, review authors caution against unclear data and reflect moderate or low quality in the studies. The highest bias refers to allocation concealment, sample size, the lack of an active control group, selection bias, and the lack of control for included confounding variables.

The levels of evidence to make the recommendations for use indicate a grade of recommendation B, where there is moderate evidence of the interventions studied [69]. Perhaps it would be possible, with greater control of confounding variables, to improve the methodological quality and the certainty of the recommendations.

### 4.1. Study Limitations

A limitation of this report is the inability to pool results and calculate effects across different systematic reviews with respect to different modalities of specific interventions and other parameters. When sufficient data were available, we planned to a perform subgroup analysis for types of psychosocial intervention, the type of assessment tool, and for studies in which some level of fatigue was an eligibility criterion for patient participation versus those in which it was not. Owing to insufficient available data, we were unable to perform these subgroup analyses. In addition, we planned to undertake subgroup analysis for the primary outcome based on aspects of the intervention that may influence its effectiveness: duration (short vs. intermediate–long), intervention delivery (group vs. individual, psychologist vs. other profession), intervention type (monodisciplinary vs. multi-disciplinary) and aim of the intervention (aimed at decreasing fatigue vs. other).

Within individual systematic reviews and meta-analyses, heterogeneity was often reported as significant, challenging the valid calculation of reported effects. There was also significant concern about the lack of data extraction and, above all, the disparity in the comparison of the results reported. To maintain the integrity of the findings, the authors argued against pooled effects and opted for providing descriptive findings.

Finally, the risks of bias detected must be pointed out. One of them is reporting bias, such as the university affiliation, the countries of study or the editorial guidelines of the journal of publication, which can condition selective reporting. The presence of incomplete outcome data can depend on the interest of the editors (attrition bias).

### 4.2. Clinical Implications

According to statistics, more than 70% of cancer patients can experience fatigue symptoms. Fatigue is a common and distressing symptom among cancer survivors. In recent years, CRF has become a major concern among the medical community. It is one of the most difficult symptoms to confront and treat for cancer patients. If fatigue is a main symptom, a specific focus of interventions on decreasing fatigue seems beneficial for patients with clinically relevant levels of fatigue. Designing interventions to specifically target fatigue is of clinical significance.

The reason for this review is the need to establish evidence for FRC reduction protocols through psychosocial interventions. However, the overall quality of the included reviews was moderate, limiting the ability to draw decisive conclusions regarding specific elements in prescribing psychological interventions for fatigue management.

Many trials present evidence supporting significant benefits of their interventions at different times of disease (before, during and after active medical treatment) and consider the type of cancer, treatment side effects or even physical deficits that can present. However, consistent comparisons are needed to establish robust and useful evidence to guide the work of healthcare providers.

Finally, the different trials did not report the impact of the intervention in the medium or long term or the continuity of positive results resulting from the intervention. In general, it is considered that these types of interventions are safe and positive in controlled settings, but we still do not have evidence regarding adherence to the practice of the different techniques learned, as well as their long-term impact. Future research should seek to better understand the long-term impact on endpoints, such as time to disease recurrence, duration of overall survival and overall mortality rates.

### 4.3. Implications for Research

Information obtained regarding review characteristics demonstrates both the strengths and weaknesses of existing research. The reviews included in this study present data of moderate or even low levels of evidence quality. However, despite the methodological limitations, the desired effects of the intervention clearly outweigh the undesired effects, resulting in a moderate GRADE recommendation.

In general, the papers included in the different reviews were RCTs and were rigorously designed, the quality assessment of the papers was Level A or B. However, the findings demonstrate the need for the publication of more detailed descriptions of complex interventions, promoting methodological strictness and transparency in the design and throughout the trial process.

Given the current state of the evidence, we recommend that researchers improve quality and reporting. The use of concealed allocation and blinded assessors is important to reduce bias, although this is not always possible in this type of intervention. Attention should also be given to the timing of assessment, the duration of the intervention to maximize benefits, longer follow-up periods and the comparison of psychosocial interventions versus usual care or attentional controls.

Further evidence is needed from high-quality trials with large samples that fully report rigorous methodological characteristics in the design stage and estimate the optimal sample size based on the existing research results, so as to ensure that the conclusions of the research carried out are sufficiently credible.

On the other hand, conducting meta-analyses with heterogeneous studies is confusing. The intervention time, frequency and cycle of self-management intervention; evaluation tools; and populations included in the literature are different, resulting in a high heterogeneity. Additional studies with homogeneous samples of cancer patients are needed. Targeting patients most in need (i.e., those reporting clinically significant levels of fatigue) to eliminate potential floor effects, would be a helpful approach in future studies.

Information abstracted regarding intervention characteristics indicated that the psychological interventions were very heterogeneous in nature. Therefore, there are still some questions that need to be answered, i.e., the optimal duration of the intervention and the best method to provide the intervention (e.g., telephone and face-to-face sessions).

Finally, the differences in scales were the sources of heterogeneity. No consensus has been reached on which instruments should be used to measure fatigue and it would be helpful to reduce the variance among outcome instruments used to measure the reduction in fatigue. According to the extracted results, the test that appears in 13% of the trials is BFI, followed by QLQ-C30 fatigue. The standardized reporting of the parameters of the different programs would be useful for investigators and would allow the aggregation of findings between the different trials, thus enabling the design of specific intervention protocols.

## 5. Conclusions

At present, psychosocial interventions specifically for fatigue are a promising type of intervention [2,27,70]. Mindfulness and cognitive therapies seem to have more evidence regarding their effectiveness. This review found a lack of clear recommendations for supporting or not supporting the use of psychosocial interventions. Most aspects of the included studies were heterogeneous and, therefore, it could not be established which types of interventions or elements were essential in reducing fatigue.

Psychosocial interventions are part of a broader portfolio of available interventions for cancer-related fatigue (e.g., physical activity and pharmacological approaches). There is a body of evidence that speaks of the importance of personal preferences towards the different techniques of this set of interventions [71,72,73], the personality of the individual and the attitude preferences regarding the significant factors impact the effectiveness of interventions. These preferences must be considered along with other factors known to underlie adherence and intention.

On the other hand, an intervention plan should be developed in the context of the known and anticipated risk of treatment-related side effects of the disease. This roadmap must be guided by a health professional who is specialized in the relevant intervention and with a solid knowledge of cancer and its treatment and sequelae in order to achieve the expected results. Leaving intervention in the hands of nonspecialized support personnel in exclusively online formats or with materials for self-management leads to low therapeutic adherence in the event of any setback.

For future research aimed at psychosocial interventions, we recommend the selection of studies with protocols for a trial, including a detailed description of the intervention and its components, published or otherwise made publicly available. It is needed to develop uniform clinical specification for practice, which helps implement interventions.

## Figures and Tables

**Table 1:** Review Characteristics.

Reference	Reviews Characteristics	Intervention GRADE	Assessment Tools	Results and Heterogeneity	Authors’ Conclusions
Abrahams, H.J.G., et al. [45], Psycho-Oncology	14 RTCs 1091 Breast cancer, 1008 Prostate cancer, 78% survivors	**Intervention**: 71% cognitive therapy and control with waiting list, GRADE—low	4	β = −0.27 [95% CI = −0.40; −0.15]). **Heterogeneity**: n/a (n/a: unspecified):	The review found statistically significant moderated effects of psychosocial interventions on fatigue. Increased effects were found for cognitive behavioural therapy and fatigue-specific interventions for patients with clinically relevant fatigue. A specific focus on decreasing fatigue seems beneficial for patients with breast cancer with clinically relevant fatigue.
Tang, Y., et al. [46] Journal of Psychosocial Oncology	9 RTCs 754 Breast cancer, 50% survivors	**Intervention**: art therapy and unspecified control, GRADE—moderate	2	(SMD = −1.90 (95% CI −2.93 to −0.87, *p* = 0.0003). **Heterogeneity**: not applicable	The review provides initial evidence to suggest that art therapy benefits patients with respect to the treatment of fatigue but is not statically significant. Additional and better-quality studies must be conducted, particularly with larger sample sizes, with greater specificity of the design of trials and interventions and a longer follow-up duration.
Huang, J., et al. [47] Clinical Rehabilitation	13 RTCs 1603 adult patients with any type of cancer, 15% survivors	**Intervention**: self-management education program with a variety of supporting online strategies and control group with psychoeducational strategies (77%), GRADE—moderate	5	Scores of CFS and MFS scales WMD = −10.15, 95% CI (−11.42, −8.89), *p* < 0.00001) **Heterogeneity**: Chi^2^ = 129.28, df = 4 (*p* < 0.00001): I^2^ = 97%, *p* < 0.01	This meta-analysis demonstrates that the internet-based self-management program for cancer-related fatigue patients, as one form of rehabilitation, can reduce the incidence of fatigue and associated symptoms among CRF patients. The long-term effects remain uncertain.
Seiler, A., et al. [48] Psycho-Oncology	8 RTCs + 1 (+6 protocols) 2264, mostly survivors of any type of cancer	**Intervention**: mostly behavioural therapies with at least one online intervention and control, GRADE—low	30	r = 0.27, 95% CI [0.1109–0.4218], *p* < 0.01). the Egger’s regression test (*p* = 0.735) and rank correlation test (*p* = 0.477) **Heterogeneity**: I^2^ = 87.46%, *p* <0.001	eHealth interventions appear to be effective for managing fatigue in cancer survivors with CRF. Analysis revealed that therapist-guided interventions were more efficacious than self-guided. The continuous development of eHealth interventions for the treatment of CRF in cancer survivors and their testing through long-term, large-scale efficacy outcome studies is encouraged.
Xu, A., et al. [49] Journal of Advanced Nursing	9 RTCs 2337, mostly adult survivors with various types of cancer	**Intervention**: mostly behavioural therapy with online support and face-to-face in control, GRADE—very low	5	SMD−0.24 (−0.39–−0.08), *p* = 0.03; χ^2^ = 17.43, df = 8 (*p* = 0.03). **Heterogeneity**: I^2^ = 54% moderate, significant in some of these subgroups	E-health-based self-management is effective for CRF. More high-quality randomized control trials are warranted to confirm these conclusions.
Corbett, T.K., et al. [50] Systematic reviews	33RTCs 4525 adult survivors, unspecified type of cancer	**Intervention**: 45% cognitive therapy and 51% waiting list in control, GRADE—moderate	15	Synthesized data narrative, unspecified data **Heterogeneity**: n/a	Eleven psychological interventions reported a significant effect. This review showed some tentative support for psychological interventions for fatigue after cancer treatment. However, as the RCTs were heterogeneous in nature and the number of high-quality studies was limited, definitive conclusions are not yet possible.
Goedendorp, M.M., et al. [51] Cochrane Database of Systematic Reviews	27RTCs 3324, patients over 16 years old in active treatment, unspecified type of cancer.	**Intervention**: mostly psychoeducational strategies and usual care control, GRADE—moderate	17	Synthesized data narrative: SMD varied between 0.17 to 1.07 **Heterogeneity**: n/a	There is limited evidence that psychosocial interventions are effective in reducing fatigue. The remaining 20 studies were regarded as not effective. At present, psychosocial interventions specifically for fatigue are a promising type of intervention. Most aspects of the included studies were heterogeneous and, therefore, it could not be established which other types of interventions or elements were essential in reducing fatigue.
Poort, H., et al. [9] Cochrane Database of Systematic Reviews	12 RTCs 535 adults with incurable cancer	**Intervention**: half cognitive therapy and half behavioural therapies and usual care control, GRADE—very low	10	SMD −0.25, 95% (CI) −0.50 to 0.00 **Heterogeneity**: T1: I^2^ = 43%, Tau^2^ = 0,08; Chi^2^ = 19.47; df = 11 (*p* = 0.05)	This review provides little evidence regarding the benefits of psychosocial interventions provided to reduce fatigue. Meta-analysis does not support the effectiveness of psychosocial interventions for reducing fatigue. The quality of the evidence in this review is very low and results of this review should be interpreted with caution.
Xie, C., et al. [52] Journal of Psychosomatic Research	15/14 RTCs 3008 adults in active treatment for any type of cancer	**Intervention**: mindfulness and usual care in control, GRADE—strong	6	SMD = −0.89, 95%CI (−1.19, −0.59), *p* < 0.001] **Heterogeneity**: I^2^ = 93% Tau ^2^ = 0.44; Chi^2^ = 298.63; df = 20 (*p* = < 0.00001)	This review indicates that mindfulness is effective for CRF management and can be recommended as a beneficial complementary therapy for CRF patients.
He, J., et al. [53] Journal of the National Medical Association	5 RTCs 700 adults for any type of cancer	**Intervention**: mindfulness and usual care in control, GRADE—low	3	SMD = −0.51,95% CI [−0.81–0.20], *p* = 0.001 **Heterogeneity**: I^2^ = 69% Tau^2^ = 0.08; Chi^2^ = 12.71; df = 4 (*p* = 0.01)	This systematic review has found that mindfulness decompression therapy can alleviate cancer-related fatigue to a certain extent. The results tend to be stable, and the grading results are shown as low-quality evidence. The mechanism needs to be further studied in the future.
Jacobsen, P.B., et al. [54] Health Psychology	41 RTCs Unspecified participants	**Intervention**: 52% cognitive therapy and usual care in control 50%, GRADE—low	unspecified	0.09 (95% CI 0.02–0.18) d_w_= 0.10, 95% CI (0.02–0.18). **Heterogeneity**: Q_w_ = 30.20 *p* < 0.05	Findings provide limited support for the use of nonpharmacological interventions to manage cancer-related fatigue. The effect size for psychological interventions, but not activity-based interventions, was statistically significant. The lack of research with heightened fatigue as an eligibility criterion is a notable weakness of the existing evidence base.

**Table 2:** Summary of the PRISMA methodological quality.

Items PRISMA	Abrahams, H.J.G., et al. [45]	Tang, Y., et al. [46]	Huang, J., et al. [47]	Seiler, A., et al. [48]	Xu, A., et al. [49]	Corbett, T.K., et al. [50]	Goedendorp, M.M., et. al. [51]	Poort, H., et al. [9]	Xie, C., et al. [52]	He, J., et al. [53]	Jacobsen, P.B., et al. [54]
1	+	+	+	+	+	+	+	+	+	+	+
2	+		+	+	+	+	+	+	+	+	+
3	+	+	+	+	+	+	+	+	+	+	+
4		+	+	+	+		+	+	+		+
5	+			+		+				+	+
6	+	+	+	+	+	+	+	+	+	+	+
7	+	+	+	+	+	+	+	+	+	+	+
8			+		+	+	+	+	+	+	+
9	+	+		+	+	+	+	+	+	+	
10		+		+	+	+	+	+	+	+	+
11	+	+	+	+	+		+	+	+	+	
12	+	+		+	+	+	+	+	+	+	+
13	+				+		+	+		+	+
14	+	+	+	+	+		+	+	+	+	+
15	+					+	+	+			
16	+	+		+		+	+	+	+	+	+
17	+	+	+		+	+	+	+	+	+	
18	+	+	+	+	+	+	+	+	+	+	+
19		+	+	+	+	+	+	+	+	+	+
20	+	+	+	+	+	+	+	+	+	+	
21		+	+	+	+		+	+	+	+	+
22		+			+	+	+	+	+	+	
23		+	+	+	+	+	+	+	+	+	+
24	+	+	+	+	+	+	+	+	+	+	+
25	+	+	+	+	+	+	+	+	+	+	+
26	+	+	+	+	+	+	+	+	+	+	+
27	+		+	+	+	+	+	+	+	+	
Total	20	21	19	22	24	22	26	26	24	25	20

+ PRISMA item completed.

## Data Availability

Not applicable.

## Appendix A. Summary of Methodological Quality and RoB Assessment in Included Studies

**Table:**

**KERRYPNX**	**Level of Evidence (Oxford)** **Level of Certainty (GRADE)**	**Authors**	**Cochrane Risk-of-Bias Tool for Randomized Trials ^1^**							**Risk-of-Bias Judgment ^2^**
			**1a**	**1b**	**2a**	**3**	**4**	**5**	**Other Bias**	
Abrahams, H.J.G., et al. [45]	Level 2B; low-quality RTC; control y follow-up. ⴲⴲⴱⴱ Low:	Arving 2007	n/a	n/a	n/a	n/a	n/a	n/a	n/a	
		Duijts 2012	n/a	n/a	n/a	n/a	n/a	n/a	n/a	
		Gellaitry 2010	n/a	n/a	n/a	n/a	n/a	n/a	n/a	
		Gielissen 2006	¿	¿	¿	¿ ^3^	ⴱ ^4^	ⴲ ^5^	ⴲ	Some concerns
		Graves 2003	n/a	n/a	n/a	n/a	n/a	n/a	n/a	
		Heiney 2003	n/a	n/a	n/a	n/a	n/a	n/a	n/a	
		Mann 2012	n/a	n/a	n/a	n/a	n/a	n/a	n/a	
		Norhouse 2005	n/a	n/a	n/a	n/a	n/a	n/a	n/a	
		Savard 2005	¿	¿	ⴱ	¿	ⴲ	ⴲ	ⴲ	Some concerns
		Van den Berg 2015	ⴲ	ⴲ	ⴱ	ⴱ	ⴲ	ⴲ	ⴲ	Low
		Chambers 2013	n/a	n/a	n/a	n/a	n/a	n/a	n/a	
		Northouse 2007	n/a	n/a	n/a	n/a	n/a	n/a	n/a	
		Goedendorp 2010	n/a	n/a	n/a	n/a	n/a	n/a	n/a	
		Johansson 2008	ⴲ	ⴲ	¿	ⴱ	ⴲ	¿	n/a	Some concerns
Tang, Y., et al. [46]	Level 2B; low-quality RTC sample sizes, specificity interventions, follow-up ⴲⴲⴲⴱ Moderate:	Xie 2006	ⴲ	ⴲ	ⴲ	ⴲ	ⴱ	¿	ⴲ	Moderate
		Gil Bar-sela 2007	ⴲ	ⴱ	ⴲ	ⴲ	ⴲ	¿	¿	Some concerns
		Wu 2010	ⴱ	ⴲ	ⴲ	¿	ⴲ	ⴱ	ⴲ	Moderate
		Lu and Hu 2010	ⴲ	ⴲ	ⴲ	ⴲ	ⴲ	¿	ⴲ	Moderate
		Zhou and LI 2011	ⴲ	ⴲ	ⴲ	ⴲ	¿	ⴲ	ⴲ	Moderate
		Chen, 2011	ⴲ	ⴲ		ⴲ	ⴲ	ⴲ	¿	Some concerns
		Zhou 2011	ⴲ	ⴱ		¿	ⴲ	ⴲ	ⴲ	Moderate
		Huang 2012	ⴲ	ⴱ		¿	¿	ⴲ	ⴲ	Some concerns
		Romito 2013		ⴲ		ⴲ	ⴲ	ⴲ	¿	Some concerns
Huang, J., et al. [47]	Level 2B; low-quality RCT. ⴲⴲⴲⴱ Moderate:	Li 2005	ⴲ	¿	ⴲ	¿	ⴲ	ⴲ	¿	Some concerns
		Zeng 2008	ⴲ	¿	¿	ⴲ	ⴲ	ⴲ	¿	Some concerns
		Wang 2010	¿	¿	¿	¿	ⴲ	ⴲ	¿	Some concerns
		Huang 2010	¿	¿	ⴲ	¿	ⴲ	ⴲ	¿	Some concerns
		Luo 2012	¿	¿	¿	¿	ⴲ	ⴲ	¿	Some concerns
		Zhang 2013	ⴲ	¿	¿	¿	ⴲ	ⴲ	¿	Some concerns
		Zhao 2014	ⴲ	¿	¿	¿	ⴲ	ⴲ	¿	Some concerns
		Liu 2016	¿	¿	¿	¿	ⴲ	ⴲ	¿	Some concerns
		Amanda P 2010	ⴲ	ⴲ	ⴲ	¿	ⴲ	ⴲ	¿	Some concerns
		Roy 2017	ⴲ	ⴲ	ⴲ	¿	ⴲ	ⴲ	¿	Some concerns
		Ding 2016	ⴱ	¿	¿	¿	ⴲ	ⴲ	¿	Some concerns
		Zhou 2017	ⴲ	¿	ⴲ	¿	ⴲ	ⴲ	¿	Some concerns
		Chen 2018	ⴲ	¿	ⴲ	¿	ⴲ		¿	Some concerns
		Purcell 2011								
		Willems 2017								
Seiler, A., et al. [48]	Level 2B; low-quality RTC. ⴲⴲⴱⴱ Low	Bantum 2014	ⴲ	¿	¿	ⴱ	ⴲ	ⴲ	-	Some concerns
		Bruggeman- Everts 2015	ⴱ	¿	ⴱ	¿	ⴲ	ⴲ	ⴲ	
		Foster 2016	ⴲ	ⴲ	ⴱ	ⴱ	ⴲ	ⴲ	ⴲ	Some concerns
		Freeman 2015	ⴲ	ⴲ	ⴱ	ⴲ	ⴲ	ⴲ	ⴱ	Low
		Galiano 2016	ⴲ	¿	ⴲ	ⴲ	ⴱ	¿	ⴱ	Low
		Rabin 2011	ⴲ	¿	ⴱ	ⴲ	ⴲ	¿	ⴱ	Some concerns
		Ritterband 2012	ⴲ	¿	ⴱ	ⴱ	ⴲ	¿	¿	Some concerns
		Willems 2016	ⴲ	ⴲ	ⴱ	¿	ⴲ	ⴲ	ⴱ	Some concerns
		Yun 2012	ⴲ	ⴲ	ⴱ	ⴱ	ⴲ	ⴲ	ⴲ	Low
										Low
Xu, A., et al. [49]	Level 2B; low-quality RTC. ⴲⴱⴱⴱ Very low	Admiraal 2017	ⴲ	ⴲ	ⴱ	ⴱ	ⴲ	¿	ⴲ	Low
		Bantum 2014	ⴲ	¿	ⴱ	ⴱ	ⴲ	ⴱ	ⴲ	Low
		Boele 2018	ⴲ	¿	ⴱ	ⴱ	ⴲ	ⴱ	ⴲ	Low
		Foster 2016	ⴲ	¿	ⴱ	ⴱ	ⴲ	ⴲ	ⴱ	Low
		Golsteijn 2018	ⴲ	ⴲ	ⴱ	ⴱ	ⴲ	ⴲ	ⴲ	Low
		Lee 1014	ⴲ	ⴲ	ⴱ	ⴱ	ⴲ	¿	ⴲ	Low
		Owen 2005	ⴲ	ⴱ	¿	¿	ⴲ	¿	ⴲ	Some concerns
		Smith 2018	ⴲ	¿	¿	¿	ⴲ	¿	ⴲ	Some concerns
		Somer 2018	¿	¿	ⴱ	ⴱ	ⴲ	ⴱ	ⴲ	Low
		Syrjala 2018	¿	¿	¿	¿	¿	¿	ⴲ	Some concerns
		Vallerand 2018	ⴲ	ⴲ	ⴱ	ⴱ	ⴲ	ⴱ	ⴲ	Low
		Van der Berg 2015	ⴲ	ⴲ	ⴱ	ⴱ	ⴲ	ⴲ	ⴲ	Low
		Willems 2017	ⴲ	¿	ⴱ	ⴱ	ⴲ	ⴱ	ⴲ	Low
		Zhang 2013	ⴲ	ⴲ	¿	ⴲ	ⴲ	¿	ⴲ	Some concerns
		Zhu 2018	ⴲ	ⴲ	ⴱ	ⴱ	ⴲ	ⴲ	ⴲ	Low
Corbett, T.K., et al. [50]	Level 2B; low-quality RTC. ⴲⴲⴲⴱ Moderate	Bantum 2014	ⴲ	¿	ⴱ	¿	ⴲ	ⴲ	ⴲ	Some concerns
		Bennett 2007	ⴲ	¿	ⴱ	ⴱ	ⴲ	ⴲ	¿	Low
		Blaess 2016	ⴲ	¿	¿	¿	ⴲ	ⴲ	¿	Some concerns
		Bower 2015	¿	¿	ⴱ	¿	ⴲ	ⴲ	¿	Some concerns
		Bruggeman-Everts 2017	ⴲ	¿	ⴱ	ⴲ	ⴱ	ⴲ	¿	Low
		Carlson 2016	ⴲ	ⴲ	¿	¿	ⴱ	¿	¿	Some concerns
		Dirksen 2008	ⴲ	¿	ⴱ	¿	ⴲ	ⴲ	ⴲ	Some concerns
		Dodds 2015	ⴲ	¿	¿	¿	ⴱ	¿	¿	Some concerns
		Dolbeault 2009	ⴲ	¿	¿	¿	ⴱ	ⴲ	ⴲ	Some concerns
		Espie 2008	ⴲ	¿	ⴱ	¿	¿	ⴲ	ⴲ	Some concerns
		Ferguson 2016	ⴲ	¿	ⴱ	ⴲ	¿	¿	¿	Some concerns
		Fillion 2008	ⴲ	¿	ⴱ	¿	ⴲ	ⴱ	ⴲ	Low
		Foster 2015	ⴲ	¿	ⴱ	ⴲ	ⴱ	ⴲ	ⴲ	Low
		Freeman 2015	¿	¿	ⴱ	¿	¿	ⴲ	ⴲ	Some concerns
		Gielissen 2006	¿	¿	¿	¿	ⴱ	ⴲ	ⴲ	Some concerns
		Heckler 2016	ⴲ	ⴲ	¿	ⴲ	ⴱ	¿	¿	Some concerns
		Hoffman 2012	ⴲ	¿	ⴱ	ⴲ	ⴱ	ⴲ	ⴲ	Low
		Johns 2014	ⴲ	ⴲ	ⴱ	¿	ⴲ	ⴲ	ⴲ	Moderate
		Lenngacher 2012	¿	¿	¿	¿	ⴲ	ⴲ	ⴲ	Some concerns
		Matthews 2014	¿	¿	ⴱ	¿	ⴲ	ⴲ	ⴲ	Some concerns
		Prinsen 2013	ⴲ	ⴲ	ⴱ	¿	ⴱ	ⴱ	ⴲ	Low
		Reeves 2017	ⴲ	¿	ⴱ	ⴲ	ⴱ	ⴲ	¿	Low
		Reich 2017	¿	¿	ⴱ	¿	ⴲ	¿	¿	Some concerns
		Reif 2012	ⴲ	ⴲ	ⴱ	ⴲ	ⴲ	ⴲ	ⴲ	Moderate
		Ritterband 2012	¿	¿	ⴱ	¿	ⴲ	ⴲ	¿	Some concerns
		Rogers 2017	ⴲ	ⴲ	ⴱ	ⴲ	ⴲ	¿	¿	Some concerns
		Sandler 2017	ⴲ	¿	ⴱ	¿	ⴲ	¿	ⴱ	Low
		Savard 2005	¿	¿	ⴱ	¿	ⴲ	ⴲ	ⴲ	Some concerns
		Van Der Lee 2012	ⴲ	ⴱ	ⴱ	¿	ⴲ	ⴱ		Low
		Van Weert 2010	ⴲ	¿	ⴱ	ⴱ	ⴲ	ⴲ	ⴲ	Low
		Willems 2016	ⴲ	¿	ⴱ	ⴲ	ⴲ	ⴲ	¿	Some concerns
		Yun 2017	ⴲ	¿	ⴱ	¿	ⴱ	ⴲ	¿	Some concerns
		Yun 2012	ⴲ	¿	ⴱ	ⴲ	ⴲ	ⴲ	ⴲ	Moderate
Poort, H., et al. [9]	Level 2B; low-quality RTC, very low-quality evidence. ⴲⴱⴱⴱ Very low	Armes 2007	ⴲ	ⴲ	¿	ⴲ	ⴲ	ⴱ		Moderate
		Barsevick 2004	¿	¿	¿	ⴲ	ⴲ	¿		Some concerns
		Barsevick 2010	¿	¿	¿	ⴲ	ⴲ	¿		Some concerns
		Bordeleau 2003	¿	ⴲ	¿	ⴲ	ⴲ	¿		Some concerns
		Bruera 2013	¿	¿	¿	ⴱ	ⴲ	ⴱ		Some concerns
		Chan 2011	¿	¿	ⴲ	ⴲ	ⴲ	¿		Some concerns
		Classen 2001	ⴲ	¿	¿	¿	ⴲ	¿		Some concerns
		Edelman 1999	ⴲ	¿	¿	ⴱ	ⴲ	¿		Some concerns
		Johansson 2008	ⴲ	ⴲ	¿	ⴱ	ⴲ	¿		Some concerns
		Savard 2006	ⴲ	¿	ⴲ	¿	ⴲ	ⴱ		Some concerns
		Sharpe 2014	ⴲ	ⴲ	ⴲ	ⴲ	ⴲ	ⴲ		Hight
		Spiegel 1981	¿	¿	¿	¿	ⴲ	ⴱ		Some concerns
		Steel 2016	ⴲ	ⴲ	ⴲ	ⴲ	¿	¿		Some concerns
		Walker 2014	ⴲ	ⴲ	ⴲ	ⴲ	ⴲ	¿		Moderate
Xie, C., et al. [52]	Level 2B; heterogeneous RTC (type of cancer, cancer stage, lost to follow-up). ⴲⴲⴲⴲ Strong	Li Q 2018	¿	¿	ⴱ	¿	ⴲ	ⴲ	ⴱ	Low
		Tang 2018	¿	¿	ⴱ	¿	ⴲ	ⴱ	ⴲ	Low
		Gao 2017	ⴲ	¿	ⴱ	¿	ⴲ	ⴲ	ⴲ	Some concerns
		Wang K a2017	¿	¿	ⴱ	ⴱ	ⴲ	ⴲ	ⴲ	Low
		Wang K b2017	¿	¿	ⴱ	ⴱ	ⴲ	ⴲ	ⴲ	Low
		Wang P 2017	ⴱ	¿	ⴱ	¿	ⴲ	ⴲ	ⴲ	Low
		Qing 2018	ⴲ	¿	ⴱ	¿	ⴲ	ⴲ	ⴲ	Some concerns
		Liu, LL 2017	ⴲ	¿	ⴱ	¿	ⴲ	ⴲ	ⴲ	Some concerns
		Reich 2014	¿	¿	ⴱ	¿	ⴲ	ⴲ	ⴲ	Some concerns
		Lengacher 2016	¿	¿	ⴱ	ⴱ	ⴲ	ⴲ	ⴱ	Low
		Reich 2017	¿	¿	ⴱ	¿	ⴲ	ⴲ	ⴲ	Some concerns
		Lengacher 2012	¿	¿	ⴱ	¿	ⴲ	ⴲ	ⴲ	Some concerns
		Hoffman 2012	ⴲ	ⴲ	ⴱ	¿	ⴱ	ⴲ	ⴱ	Low
He, J., et al. [53]	Level 2B; heterogeneous RTC (type of cancer, cancer stage, lost to follow-up. ⴲⴲⴱⴱ Low	Cao 2016	U	U	U	U	L	L	L	B
		Tang 2018	U	U	U	U	L	L	L	B
		Johns 2015	L	L	L	L	L	L	L	A
		Johns 2016	L	L	L	L	L	L	L	A
		Lengacher 2016	L	U	U	U	L	L	L	B
Goedendorp, M.M., et al. [51] Internal validity (vanTulder,97). Cochrane			Jadad Internal validity							
	Level 2B; low quality RTC ⴲⴲⴲⴱ Moderate	Armes 2007	ⴲ	ⴱ		ⴱ	2	4.5		
		Barsevick 2004	ⴲ	ⴱ		ⴱ	1	4.5		
		Cohen 2007	ⴲ	ⴱ		ⴲ	1	4.5		
		Forester 1985	ⴲ	ⴱ		ⴱ	1	2.5		
		Ream 2006	ⴲ	ⴲ		ⴱ	3	4		
		Spiegel 1981	ⴲ	ⴱ		ⴱ	1	2.5		
		Yates 2005	ⴲ	ⴲ		ⴱ	3			

^1^ Bias Domains. (1) Selection bias: (a) bias arising from the randomisation process; (b) allocation concealment. (2) Performance bias: (a) blinding of participants and personnel; (b) bias due to de-viations from intended interventions. (3) Detection bias: blinding of outcome assessment. (4) At-trition bias: (a) bias due to missing outcome data; (b) bias in the measurement of the outcome. (5) Reporting bias: bias in selection of the reported result. ^2^ Overall bias: risk-of-bias judgment (low/high/some concerns). ^3^ Unclear risk of bias (Cochrane). ^4^ High risk of bias (Cochrane). ^5^ Low risk of bias (Cochrane).

## Appendix B. Review Characteristics

**Table:**

**Reference**	**Delivery by**	**Specific Fatigue ^1^** **Frequency**	**Delivery Mode ^2^** **Type of Delivery ^3^**	**Outcome Measure**
Abrahams, H.J.G., et al. [45]	Psychologic (7); nurse (4); social worker (1); PhD candidate (1); group therapist (1); unspecified (1)	y (2); n (12) 3–13 sessions	(5); (5); (2); n/a (2) (9); (2); = (2); + = (1)	5 (QLQ-C30); SF-36; POMS fatigue; CIS fatigue; MFI global fatigue
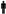
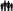
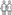
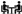
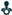
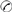
Tang, Y., et al. [46]	Unspecified	Unspecified Unspecified	n/a n/a	2 (BFI; RPFS-C)
Huang, J., et al. [47]	Doctor, nurse, other professional; unspecified number	y (8); n (5) 1 week–6 months	(13) (13); = n/a	8 (BFI) 5 (CFS; MFS); FACT-G; FACT-B;
Seiler, A., et al. [48]	Unspecified	y (9); n (6) 6–48 weeks	(15); (6), (9); n/a (6)	4 (CIS); SIP; 2 (BSI-18); 4 (EORTC - QLQ - C30); 4 (BFI); WHIRS; PHQ-8; FFQ; 4 (HADS); 2 (PFS-R); QLACS; 2 (PSEFSM); 2 (SC-SES); 2 (FACT-G); PWI; 2 (PHQ-9); FACIT-F; SF-36; PSQI; BPI; PWI; PAR; POMS; ISI; MFSI-SF; WAI; FSS; ECSI; MNA; MOS-SS
Xu, A., et al. [49]	Unspecified programme developer	y (1); n (14) 4 weeks–6 months	(15) (13); = (2)	4 (BFI); 3 (CIS); 2 (FACIT - F); PROMIS; FSI
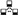
Corbett, T.K., et al. [50]	Psychologist (12); nurse (6); cancer survivor (2); therapist (5); Master’s student (4); coach (3); doctor (2); other professional (1); social workers (1); PhD candidate (1); unspecified (6)	Unspecified 4 weeks–6 months	(16); (21); (7); = (5); (21)	3 (BFI); SCFS; 2 (FACT-F); 5 (FSI); 4 (POMS); SF-12; 3 (EORTC-F); 3 (MFI); 3 (FACIT-F); 3 (CIS-f); MDASI; PFS; FAQ; MFSI-SF; FSS
Goedendorp, M.M., et al. [51]	Nurse (11); others professional (9); doctor (1); therapists (2); psychologist (1); social worker (2); unspecified (1)	y (5); n (22) 3 sessions–1 year/weekly	(17); (10) (20); = (3); + = (3); O(1);	4 (VAS-f); 6 (EORTC QLQ-C30); 2 (MFI); 12 (POMS); SCFS; GFS; LASA; STAI; SDS; FSI; SADS; SES; 2 (FACT-F); FACIT-F; SF-36; PFS; NFRS
Poort, H., et al. [9]	Nurse (4); others professional (2); therapist (2)	y (6); n (8) 2 weeks–12 months	(6); (8); (4); = (3); + = (4); (1)	5 (POMS5); 4 (VAS); 5 (EORTC QLQ-C30); FACIT; PFS; MFI; FACT-F; SCFS; GFS; ESAS
Xie, C., et al. [52]	Coach (15)	n (15) 6–8 weeks	(15) n/a (15)	6 (PFS-R); 3 (CFS); FSS; 2 (DASI); 2 (FSI); POMS
He, J., et al. [53]	Unspecified (5)	Unspecified (5) 6–8 weeks	(5) n/a (5)	CFS; PFS; 3 (FSI)
Jacobsen, P.B., et al. [54]	Unspecified (24)	y (5); n (19) 1 session–1 year	(7); (4); n/a (30) (2); = (2); O (2); n/a (18)	n/a

^1^ Specific fatigue: Y = specific fatigue program; N = nonspecific fatigue program; n/a = unspecified. ^2^ Delivery mode:  = individual;  = group;  =couple; n/a = unspecified. ^3^ Type of delivery:  = self-guided;  = telephone;  = face to face;  online; O = others; n/a = unspecified.
